# Correlations between Age, Pain Intensity, Disability, and Tactile Acuity in Patients with Chronic Low Back Pain

**DOI:** 10.1155/2022/2907009

**Published:** 2022-03-25

**Authors:** Juan Wang, Kangyong Zheng, Jinlong Wu, Rui Wang, Xiao Zhuang, Xueqiang Wang

**Affiliations:** ^1^Department of Rehabilitation Medicine, Changzhou Geriatric Hospital Affiliated to Soochow University, Changzhou 213011, China; ^2^Department of Rehabilitation Medicine, Changzhou No. 7 People's Hospital, Changzhou 213011, China; ^3^Department of Sport Rehabilitation, Shanghai University of Sport, Shanghai 200438, China; ^4^Department of Physical Education, Shenzhen University, Shenzhen 518060, China; ^5^Department of Rehabilitation Medicine, Shanghai Shangti Orthopaedic Hospital, Shanghai 200438, China

## Abstract

**Objective:**

Chronic low back pain is an overwhelming problem for a wide range of people and leads to tactile acuity deficits. We aimed to investigate the correlations among age, pain severity, disability, and tactile acuity in patients with chronic low back pain by using multiple tactile acuity tests.

**Methods:**

A total of 58 participants (36.40 ± 14.95 years) with chronic low back pain were recruited, and two-point discrimination, point-to-point test, and two-point estimation were performed on their painful low back areas. The correlations between age, pain intensity, disability, and tactile acuity were characterized with Pearson's correlation coefficients. Subgroup analyses according to the median values of age, pain intensity, and disability were used to compare the intergroup difference in tactile acuity.

**Results:**

Results illustrated significant negative associations among age, pain intensity, disability, and tactile acuity. Subgroup analyses revealed that patients with below-the-median values of age, pain intensity, and disability had better performance in tactile acuity tests than those with above-the-median values.

**Conclusion:**

This study indicated that tactile acuity was negatively associated with age, pain intensity, and disability in young patients with chronic low back pain.

## 1. Introduction

Tactile acuity, a perception function of touch stimulation, includes central and peripheral neural mechanisms [1]. The primary sensory cortex (S1), the area responsible for tactile acuity, modulates touch signals that are transmitted from the Merkel disc, a cutaneous serotonergic synapse [2, 3]. Chronic pain is accompanied by cortical alterations that can be reflected by a reduction in tactile acuity [4]. Tactile acuity tests are regularly used to provide insights into the tactile abilities of healthy and diseased populations and to explore pain-related somatosensory changes indirectly. Typical tactile acuity tests include force detection with calibrated monofilaments, which are simple but fragile and can be distorted after multiple uses, and body image drawings that preclude quantitative analysis [5–7]. Two-point discrimination (TPD), two-point estimation (TPE), and point-to-point test (PTP) are other classical spatial discrimination tests that provide reliable and repeatable measures [8–10]. Our previous study confirmed that TPD, PTP, and TPE tests had good intrarater reliability and moderate-to-good interrater reliability in patients with chronic low back pain (CLBP) at different ages. These three measurements have good clinical applicability [8]. Although other tests, such as electroencephalography and functional magnetic resonance imaging, directly reflect related cortex changes, they are not conducive to clinical promotion because of their complicated operation and high cost [8, 11, 12].

Tactile acuity is known to diminish with age in healthy populations due to mechanoreceptor loss [13–15]. Tactile acuity deficits have been confirmed as characteristics of chronic pain conditions, including arthritis, complex regional pain syndrome, and frozen shoulder, which are accompanied by the cortical reorganization of S1 [4, 16]. Studies proved that exercise can improve pain [17–19]. Motor control training combined with tactile acuity training can improve patients' motor control and sensory discrimination abilities [20]. Additionally, Luomajoki and Moseley found that the threshold of low back TPD was greater in patients with CLBP than in healthy controls and that a larger TPD threshold at the back relates to worse voluntary lumbopelvic control [21]. It indicates that tactile acuity training has the potential for sensory rehabilitation of CLBP. Extensive evidence suggests that CLBP is accompanied with cortical changes, altering S1 representation by approximately 2 cm or by neurons at different levels and regions [21–23]. Some studies show that the extent of cortical change is linked to pain intensity [23–26]. These studies indicate that potential associations exist among age, pain severity, disability, and tactile acuity. However, these associations are incompletely understood because most related studies were limited to TPD testing, which is prevalent but flawed [27, 28]. Depending on TPD alone seems insufficient for quantifying tactile acuity reliably [9, 29].

CLBP is an overwhelming problem in a wide range of people and leads to sensory deficits. Although a preliminary study demonstrated that the intra and interrater reliabilities of TPD, PTP, and TPE on the level of the fifth lumbar vertebrae (L5) are moderate to good, it did not describe in detail the correlations between tactile acuity and its influencing factors, such as age, pain severity, and disability, therefore leaving the question open for investigation [8]. Thus, this study aimed to investigate the correlations among age, pain severity, disability, and tactile acuity in patients with chronic low back pain by using multiple tactile acuity tests.

## 2. Methods

### 2.1. Study Design

The study design in this research is cross-sectional.

### 2.2. Setting

This study was performed in Shanghai Shangti Orthopaedic Hospital from December 2018 to May 2019. And the data collection was completed by the author J Wang, reducing the potential for error caused by different operators.

### 2.3. Participants

All the participants were recruited through posters from Shanghai University of Sport, Shanghai Shangti Orthopaedic Hospital, and nearby communities. As a result, 58 participants with CLBP (36.40 ± 14.95 years, 168.47 ± 7.95 cm, and 65.84 ± 10.52 kg) completed the study. The eligibility criteria included [1] between the ages of 18 and 65 years; [2] the persistent presence of CLBP lasting at least 50% of the time during the past 6 months [3, 8]; unilateral lumbar pain covering L5; and [4] normal upper limb motor function. The exclusion criteria were [1] a state of serious psychiatric conditions or cognitive impairment that would interfere with the understanding of the study procedures or [2] spinal surgery history. The study was approved by the research ethics committees of Shanghai University of Sport and was registered in the Chinese Clinical Trial Registry (ChiCTR2100043940). Every participant signed an informed consent form in advance.

### 2.4. Data Measurement

We included the following outcome measures before the experiment: age, pain intensity, and disability. For pain intensity, the participants reported their general pain, maximum pain, and pain unpleasantness during the past 3 months. A numerical rating scale that ranged from 0 (“no pain”) to 10 (“the worst pain”) was used as the scoring standard to quantify pain intensity. Disability was measured by using the Oswestry Disability Index (ODI) and the Roland–Morris Disability Questionnaire (RMDQ), which both have good validity and reliability [30–32]. High scores on the ODI and RMDQ indicate severe dysfunction.

### 2.5. Tactile Acuity Measurement

The participants were positioned comfortably in a prone position with their lower back exposed. TPD, PTP, and TPE were performed on every participant along the horizontal line at the L5 level of the painful side by using caliper rulers (Powerfix, digital caliper: Z22855; precision: 0.01 mm).

In TPD, the minimum distance to perceive two points instead of one was measured. The TPD test was performed in the order of ascending (A-TPD) or descending (D-TPD) methods in accordance with previously published research [8]. In A-TPD, the caliper was initially started with a 20 mm separation between its tips and increased by 5 mm increments until the participant reported only one point. The A-TPD threshold was repeated 3 times to obtain the average ascending value, which was the initial distance of the caliper tips in the subsequent D-TPD test. The D-TPD threshold was also repeatedly confirmed three times.

In PTP, a caliper was used to measure the distance between the stimulus point of the examiner and the verification point of the participant in accordance with a previous study [8, 10]. The examiner lightly and randomly touched one of the stimulus points. Then, the participants were instructed to point out the locations with a pen as accurately as possible. The distance between the stimulus point touched by the examiner and the one touched by the participant was measured three times for the PTP.

TPE, a novel diagnostic measurement for tactile acuity, compares the stimulation distance with the distance estimated by the participant and was performed in accordance with a previous study [8]. In this session, two mechanical calipers were utilized: one by the examiner and one by the participant. The examiner performed a tactile test along the L5 horizontal level with a 120 mm initial stimulation distance. The participants were instructed to match the distance that they felt by using another caliper that only displayed the backside. The TPE threshold was defined as the difference between 120 mm and the distance matched by the participant. This test was also repeated three times, and the average TPE value was obtained.

The abovementioned tests were conducted in random order. Low A-TPD, D-TPD, PTP, or TPE thresholds indicate great tactile acuity. The intra and interrater reliabilities of TPD, PTP, and TPE on the low back area are moderate to good [8, 10].

### 2.6. Sample Size Calculation

With the type I error at 5%, the statistical power at 80% and the effect size at 0.36 (medium magnitude), 55 subjects at least should be included in this study, which was calculated by G^*∗*^Power 3.1 software [33].

### 2.7. Statistical Analysis

SPSS version 20.0 (SPSS 3 Inc., Chicago, IL, USA) was used for statistical analyses. All data in this study were normal distributed. The associations among age, pain intensity, disability, and tactile acuity (i.e., A-TPD, D-TPD, PTP, and TPE) were characterized by using Pearson's correlation coefficients. The r value was calculated according to the results of previous research (nonexistent, 0.00–0.09; small, 0.10–0.29; medium, 0.30–0.49; large, 0.50–0.69; very large, 0.70–0.89; nearly perfect, 0.90–0.99; and perfect, 1.00) [34]. For subgroup analyses, the 58 participants were equally divided into the above and belowmedian groups in terms of age (median = 32 years), general pain intensity (median = 4), maximum pain (median = 5), pain unpleasantness (median = 7), ODI (median = 21.5), and RMDQ (median = 6), respectively. Independent-sample *T* test was applied to compare the intergroup difference of tactile acuity. *P* < 0.05 was statistically significant.

## 3. Results

### 3.1. Demographics and Clinical Characteristics

The characteristics of the participants with CLBP are summarized in Table 1. The median values of age, maximum pain, general pain, pain unpleasantness, ODI, and RMDQ were 32 years, 5, 4, 7, 21.5, and 6, respectively.

### 3.2. Age and Tactile Acuity

The results of Pearson's correlation analysis between age and tactile acuity are presented in Table 2. Medium-to-large positive correlations were observed for age and tactile acuity. The results indicated that age had medium-positive correlations with D-TPD (*r* = 0.345, *P*=0.008) and A-TPD (*r* = 0.42, *P*=0.001) and had large-positive correlations with PTP (*r* = 0.617, *P* < 0.001) and TPE (*r* = 0.611, *P* < 0.001).

### 3.3. Maximum Pain and Tactile Acuity

The results of Pearson's correlation analysis between maximum pain and tactile acuity are given in Table 2. Medium-to-large positive correlations were observed for maximum pain and tactile acuity. The results indicated that maximum pain had medium positive correlations with D-TPD (*r* = 0.331, *P*=0.011), A-TPD (*r* = 0.385, *P*=0.003), and TPE (*r* = 0.449, *P* < 0.001) and had a large-positive correlation with PTP (*r* = 0.565, *P* < 0.001).

### 3.4. General Pain and Tactile Acuity

The results of Pearson's correlation analysis between general pain and tactile acuity are depicted in Table 2. Medium-to-large positive correlations were observed for general pain and tactile acuity. The results indicated that general pain had medium-positive correlations with TPD (*r* = 0.355, *P*=0.006), A-TPD (*r* = 0.387, *P*=0.003), and TPE (*r* = 0.467, *P* < 0.001) and had a large positive correlation with PTP (*r* = 0.598, *P* < 0.001).

### 3.5. Pain Unpleasantness and Tactile Acuity

The results of Pearson's correlation analysis between pain unpleasantness and tactile acuity are provided in Table 2. Medium-to-large positive correlations were observed for pain unpleasantness and tactile acuity. The results indicated that pain unpleasantness had medium-positive correlations with D-TPD (*r* = 0.314, *P*=0.016), A-TPD (*r* = 0.408, *P*=0.001), and TPE (*r* = 0.48, *P* < 0.001) and had a large-positive correlation with PTP (*r* = 0.572, *P* < 0.001).

### 3.6. ODI and Tactile Acuity

The results of Pearson's correlation analysis between ODI and tactile acuity are illustrated in Table 2. Medium-to-large positive correlations were observed for ODI and tactile acuity. The results indicated that ODI had medium-positive correlations with D-TPD (*r* = 0.387, *P*=0.003) and A-TPD (*r* = 0.389, *P*=0.003) and had large-positive correlations with PTP (*r* = 0.597, *P* < 0.001) and TPE (*r* = 0.573, *P* < 0.001).

### 3.7. RMDQ and Tactile Acuity

The results of Pearson's correlation analysis between RMDQ and tactile acuity are given in Table 2. Medium-to-large positive correlations were observed for RMDQ and tactile acuity. The results indicated that RMDQ had medium-positive correlations with D-TPD (*r* = 0.314, *P*=0.016) and A-TPD (*r* = 0.41, *P*=0.001) and had large-positive correlations with PTP (*r* = 0.601, *P* < 0.001) and TPE (*r* = 0.542, *P* < 0.001).

### 3.8. Subgroup Analysis Results

**Figure 1:**
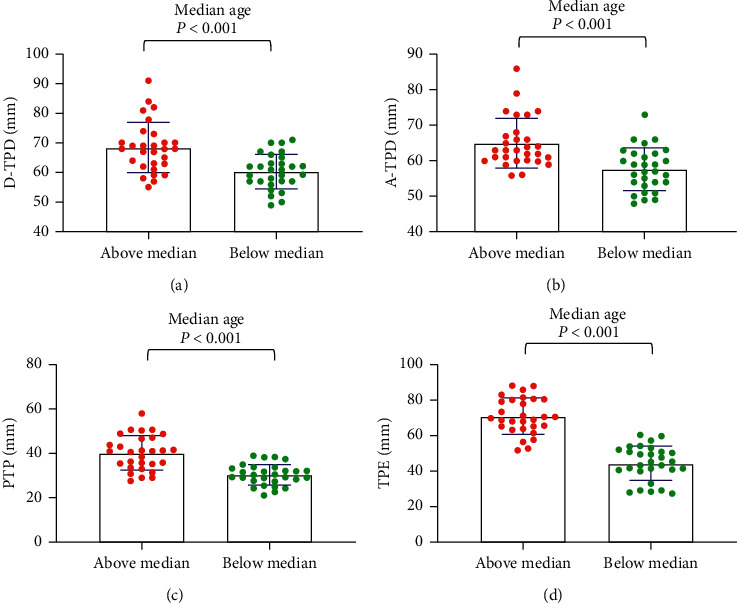
Tactile acuity for median age in the abovemedian group versus the belowmedian group. (a) D-TPD, (b) A-TPD, (c) PTP, and (d) TPE. The median of age was 32 years. D-TPD = two-point discrimination test performed in a descending manner; A-TPD = two-point discrimination test performed in an ascending manner; PTP = point-to-point test; TPE = two-point estimation.

**Figure 2:**
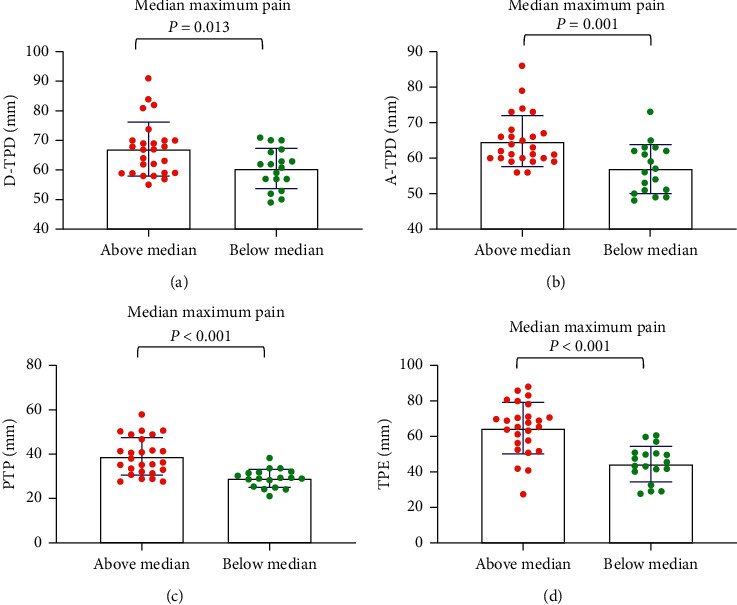
Tactile acuity for median maximum pain in the abovemedian group versus the belowmedian group. (a) D-TPD, (b) A-TPD, (c) PTP, and (d) TPE. The median of maximum pain was 5. D-TPD = two-point discrimination test performed in a descending manner; A-TPD = two-point discrimination test performed in an ascending manner; PTP = point-to-point test; TPE = two-point estimation.

**Figure 3:**
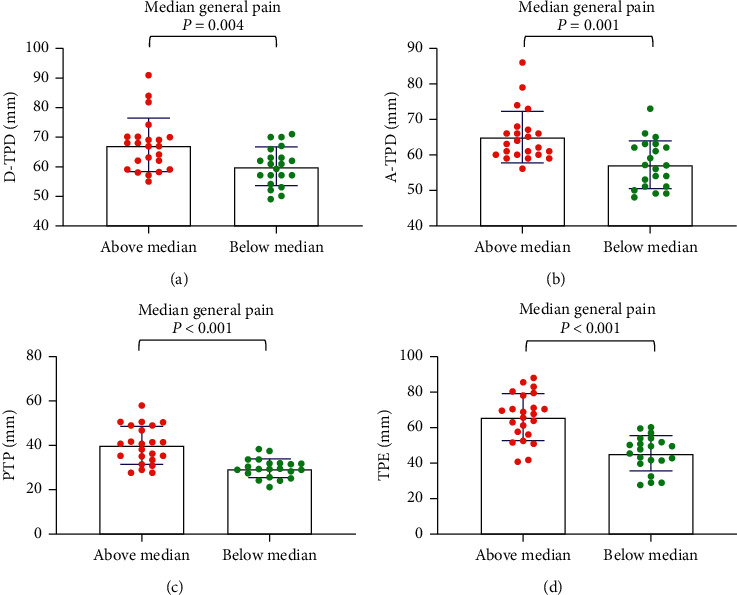
Tactile acuity for median general pain in the abovemedian group versus the belowmedian group. (a) D-TPD, (b) A-TPD, (c) PTP, and (d) TPE. The median of general pain intensity was 4. D-TPD = two-point discrimination test performed in a descending manner; A-TPD = two-point discrimination test performed in an ascending manner; PTP = point-to-point test; TPE = two-point estimation.

**Figure 4:**
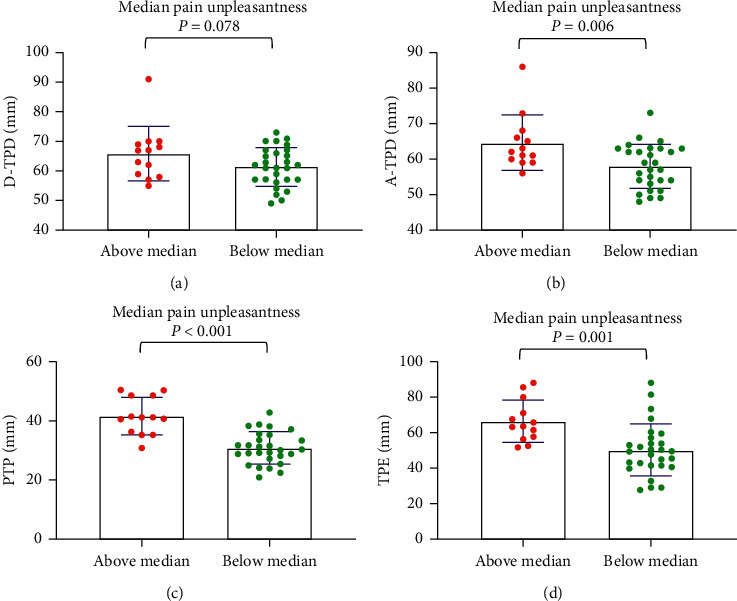
Tactile acuity for median pain unpleasantness in the abovemedian group versus the belowmedian group. (a) D-TPD, (b) A-TPD, (c) PTP, and (d) TPE. The median of pain unpleasantness was 7. D-TPD = two-point discrimination test performed in a descending manner; A-TPD = two-point discrimination test performed in an ascending manner; PTP = point-to-point test; TPE = two-point estimation.

**Figure 5:**
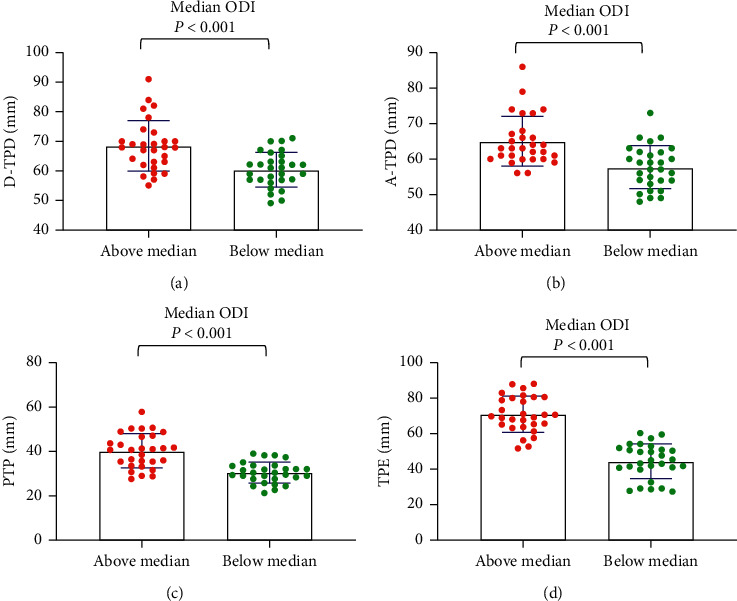
Tactile acuity for median ODI in the abovemedian group versus the belowmedian group. (a) D-TPD, (b) A-TPD, (c) PTP, and (d) TPE. The median of ODI was 21.5. D-TPD = two-point discrimination test performed in a descending manner; A-TPD = two-point discrimination test performed in an ascending manner; PTP = point-to-point test; TPE = two-point estimation; ODI = Oswestry Disability Index.

**Figure 6:**
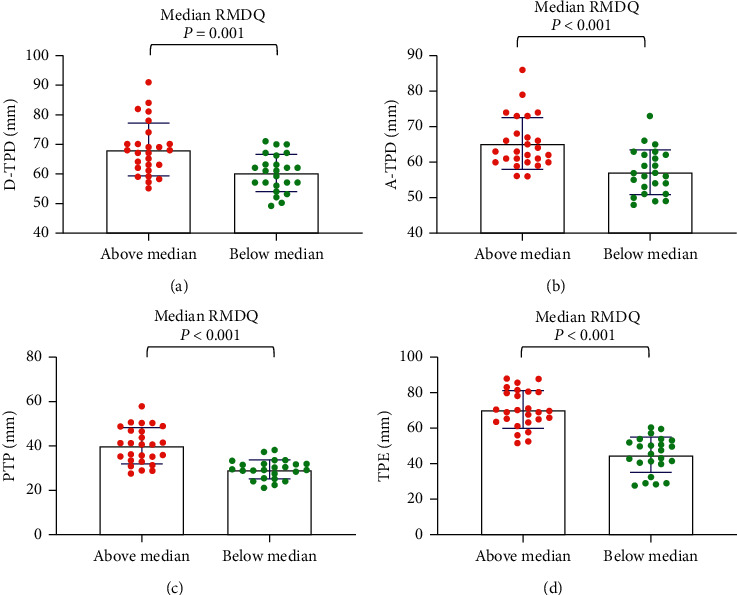
Tactile acuity for median RMDQ in the abovemedian group versus the belowmedian group. (a) D-TPD, (b) A-TPD, (c) PTP, and (d) TPE. The median of RMDQ was 6. D-TPD = two-point discrimination test performed in a descending manner; A-TPD = two-point discrimination test performed in an ascending manner; PTP = point-to-point test; TPE = two-point estimation; RMDQ = Roland-Morris Disability Questionnaire.

For the median age, the tactile acuities of the abovemedian groups were significantly worse than those of the belowmedian groups (*P* < 0.001) (Figure 1). For maximum pain, general pain, and pain unpleasantness, the tactile acuities of the abovemedian groups were significantly worse than those of the belowmedian groups (*P* < 0.05) (Figures 2–4). However, the D-TPD for median pain unpleasantness (*P*=0.078) was close only to marginal significance. For ODI and RMDQ, the tactile acuities of the abovemedian groups were significantly worse than those of the belowmedian groups (*P* ≤ 0.001) (Figures 5 and 6).

## 4. Discussion

We assessed the associations among age, pain severity, disability, and tactile acuity in CLBP. Our results showed that tactile acuity was significantly associated with the abovementioned indicators and that tactile acuity tended to be better in those who were younger, had lower pain severity, and had lower CLBP-related disability than in other participants.

Consistent with the results of Falling and Mani [35], our results confirmed that age demonstrated significant negative correlations with tactile acuity in CLBP. Similarly, other investigations on the effect of aging on perception have also shown that spatial acuity saliently declines with age [36, 37]. The well-documented loss of tactile acuity with age can be explained by a range of potential mechanisms, such as mechanoreceptor loss and high cortical excitability [36, 38]. Age is known to affect the mechanical, physical, and neurophysiological properties of the skin (e.g., the detection, transmission, or interpretation of passive sensory stimulation) [14]. By using electrical median nerve stimulation, Lenz et al. confirmed that intracortical inhibition in human SI significantly declines in the elderly and that the significant age-related enhancement in cortical excitability is linked to acuity deterioration [38]. Thus, for older patients with chronic low back pain, the influence of age on tactile acuity should be recognized and interventions to interfere with age-related changes of perception, such as modulation of cortical excitability [38], should be emphasized.

Adamczyk et al. stated that “the greater the intensity of pain, the worse the tactile acuity” [39]. Our study also found that pain severity, in terms of maximum pain, general pain, and pain unpleasantness, had an overall negative effect on tactile acuity, although in the above-median group, pain unpleasantness simply exhibited a trend of worsening D-TPD. Adamczyk et al. were the first to demonstrate that nociceptive pain itself is the contributor of tactile acuity changes in chronic and acute low back pain [39]. Some studies have found that an alteration in tactile acuity is linked to the duration and severity of suffering pain [9, 40, 41]. The touch-gate theory, which is similar to the pain gate-control theory, has been used to explain tactile acuity deterioration, and sensory thresholds have been reported to increase when suffering noxious stimulation [39, 42]. It was found that the extent of cortical reorganization has been associated to both pain severity and a reduction in tactile acuity [24, 26]. Therefore, this suggests a potential response of tactile acuity to pain recovery [43].

Our results showed that tactile acuity in the back area of those with CLBP was significantly associated with low-back function as reflected by ODI and RMDQ. That is, participants with less low-back dysfunction (below-median groups of ODI and RMDQ) were expected to have better tactile sensitivity and discrimination than the other participants. It has been confirmed that tactile acuity relates to voluntary lumbopelvic control, and weak proprioceptive acuity is a contributor to disability [21]. Similarly, previous studies have confirmed the association of tactile acuity and hand function [37, 44, 45]. For example, people with knee or hand osteoarthritis have impaired spatial sensitivity and experience great difficulties in tasks requiring fine and dexterous manipulations [40, 46]. Future studies are also expected to examine whether interventions targeting weak tactile acuity can effectively improve low-back function in those with CLBP.

Our study has several strengths. First, the multidimensional correlation of tactile acuity in CLBP was evaluated in terms of age, pain severity, and disability. Second, tactile acuity was evaluated in detail by using a succession of tests. As a result, we obtained relatively similar results with different tests, thus enhancing the persuasiveness of the conclusion. Third, our study provided robust and novel evidence concerning associations among age, pain severity, disability, and tactile acuity via subpopulation analyses of median values.

This study also has several limitations. In this experiment, the participants had a limited age range (36.4 ± 14.95 years) and were mainly young adults. The data on children and the elderly were lacking. Therefore, demographic differences in these unmeasured populations may still exist. Another limitation is that all our test methods were peripheral test methods that cannot directly reflect structural and functional cortical reorganization in patients. Thus, further studies can be carried out by using neuroimaging techniques, such as electroencephalography and functional magnetic resonance imaging, to obtain further explanations. In addition, other body factors, such as body mass index and emotional state, may contribute to the difference in tactile acuity, which should also be confirmed in subsequent studies. Our study only used bivariate estimation without adjusting the abovementioned covariates. We hope that future studies will use multiple linear regression analysis to adjust for confounding factors.

## 5. Conclusions

In conclusion, our findings confirmed negative associations among age, pain severity, disability, and tactile acuity in patients with CLBP. Severe CLBP was associated with worsening tactile acuity. Specifically, CLBP patients with advanced age, severe pain, and severe dysfunction may experience a significant deterioration in tactile acuity.

## Figures and Tables

**Table 1:** Participant characteristics.

	Total (*n* = 58)
Age (years)	36.40 (14.95)
Height (cm)	168.47 (7.95)
Weight (kg)	65.84 (10.52)
BMI (kg/m^2^)	23.18 (2.59)
Waistline (cm)	88.50 (15.13)
D-TPD	64.36 (8.30)
A-TPD	61.33 (7.44)
PTP	35.30 (8.06)
TPE	57.67 (16.62)
NRS	
Maximum pain	5.12 (1.29)
General pain	4.02 (1.29)
Pain unpleasantness	6.50 (1.19)
Disability	
ODI	20.93 (7.25)
RMDQ	6.97 (3.37)

Notes: ^*∗*^Values as mean (standard deviation); D-TPD = two-point discrimination test performed in a descending manner; A-TPD = two-point discrimination test performed in an ascending manner; PTP = point-to-point test; TPE = two-point estimation; BMI = body mass index; NRS = Numerical Rating Scale; ODI = Oswestry Disability Index; RMDQ = Roland-Morris Disability Questionnaire.

**Table 2:** The correlations among age, pain severity, disability, and tactile acuity.

	D-TPD	A-TPD	PTP	TPE
Age	0.345 (0.008)	0.420 (0.001)	0.617 (<0.001)	0.611 (<0.001)
Maximum pain	0.331 (0.011)	0.385 (0.003)	0.565 (<0.001)	0.449 (<0.001)
General pain	0.355 (0.006)	0.387 (0.003)	0.598 (<0.001)	0.467 (<0.001)
Pain unpleasantness	0.314 (0.016)	0.408 (0.001)	0.572 (<0.001)	0.48 (<0.001)
ODI	0.387 (0.003)	0.389 (0.003)	0.597 (<0.001)	0.573 (<0.001)
RMDQ	0.314 (0.016)	0.41 (0.001)	0.601 (<0.001)	0.542 (<0.001)

Notes: ^*∗*^Values as r (*P*) of Pearson's correlation coefficients; D-TPD = two-point discrimination test performed in a descending manner; A-TPD = two-point discrimination test performed in an ascending manner; PTP = point-to-point test; TPE = two-point estimation; ODI = Oswestry Disability Index; RMDQ = Roland-Morris Disability Questionnaire.

## Data Availability

The data that support the findings of this study are available from the corresponding author upon reasonable request.

## References

[1] Tamura Y., Hoshiyama M., Inui K., Kakigi R. (2003). Central mechanisms for two-point discrimination in humans. *Neuroscience Letters*.

[2] Brown P. B., Koerber H. R., Millecchia R. (2004). From innervation density to tactile acuity. *Brain Research*.

[3] Chang W., Kanda H., Ikeda R., Ling J., DeBerry J. J., Gu J. G. (2016). Merkel disc is a serotonergic synapse in the epidermis for transmitting tactile signals in mammals. *Proceedings of the National Academy of Sciences of the United States of America*.

[4] Catley M. J., O’Connell N. E., Berryman C., Ayhan F. F., Moseley G. L. (2014). Is tactile acuity altered in people with chronic pain? a systematic review and meta-analysis. *Journal of Pain*.

[5] Haloua M. H., Sierevelt I., Theuvenet W. J. (2011). Semmes-weinstein monofilaments: influence of temperature, humidity, and age. *Journal of Hand Surgery*.

[6] Moseley L. G. (2008). I can’t find it! Distorted body image and tactile dysfunction in patients with chronic back pain. *Pain*.

[7] Nishigami T., Mibu A., Osumi M. (2015). Are tactile acuity and clinical symptoms related to differences in perceived body image in patients with chronic nonspecific lower back pain?. *Manual Therapy*.

[8] Wang J., Chen C., Peng M. (2020). Intra- and inter-rater reliability of three measurements for assessing tactile acuity in individuals with chronic low back pain. *Evidence-Based Complementary and Alternative Medicine*.

[9] Adamczyk W. M., Sługocka A., Mehlich K., Saulicz E., Luedtke K. (2019). Preliminary validation of a two-point estimation task for the measurement of sensory dissociation in patients with chronic low back pain. *Pain Medicine*.

[10] Adamczyk W., Sługocka A., Saulicz O., Saulicz E. (2016). The point-to-point test: a new diagnostic tool for measuring lumbar tactile acuity? Inter and intra-examiner reliability study of pain-free subjects. *Manual Therapy*.

[11] Diers M., Koeppe C., Diesch E. (2007). Central processing of acute muscle pain in chronic low back pain patients: an EEG mapping study. *Journal of Clinical Neurophysiology*.

[12] Kregel J., Meeus M., Malfliet A. (2015). Structural and functional brain abnormalities in chronic low back pain: a systematic review. *Seminars in Arthritis and Rheumatism*.

[13] Aimonetti J.-M., Deshayes C., Crest M., Cornuault P.-H., Weiland B., Ribot-Ciscar E. (2019). Long term cosmetic application improves tactile discrimination in the elderly; a new psychophysical approach. *Frontiers in Aging Neuroscience*.

[14] Skedung L., El Rawadi C., Arvidsson M. (2018). Mechanisms of tactile sensory deterioration amongst the elderly. *Scientific Reports*.

[15] Jimenez-Andrade J. M., Mantyh W. G., Bloom A. P. (2012). The effect of aging on the density of the sensory nerve fiber innervation of bone and acute skeletal pain. *Neurobiology of Aging*.

[16] Breckenridge J. D., McAuley J. H., Ginn K. A. (2020). Motor imagery performance and tactile spatial acuity: are they altered in people with frozen shoulder?. *International Journal of Environmental Research and Public Health*.

[17] Peng M.-S., Wang R., Wang Y.-Z. (2022). Efficacy of therapeutic aquatic exercise vs physical therapy modalities for patients with chronic low back pain. *JAMA Network Open*.

[18] Wu B., Zhou L., Chen C., Wang J., Hu L., Wang X. (2021). Effects of exercise-induced hypoalgesia and its neural mechanisms. *Medicine and Science in Sports and Exercise*.

[19] Zheng K., Chen C., Yang S., Wang X. (2021). Aerobic exercise attenuates pain sensitivity: an event-related potential study. *Frontiers in Neuroscience*.

[20] Gutknecht M., Mannig A., Waldvogel A., Wand B. M., Luomajoki H. (2015). The effect of motor control and tactile acuity training on patients with non-specific low back pain and movement control impairment. *Journal of Bodywork and Movement Therapies*.

[21] Luomajoki H., Moseley G. L. (2011). Tactile acuity and lumbopelvic motor control in patients with back pain and healthy controls. *British Journal of Sports Medicine*.

[22] Schmidt-Wilcke T., Leinisch E., Gänssbauer S. (2006). Affective components and intensity of pain correlate with structural differences in gray matter in chronic back pain patients. *Pain*.

[23] Flor H., Braun C., Elbert T., Birbaumer N. (1997). Extensive reorganization of primary somatosensory cortex in chronic back pain patients. *Neuroscience Letters*.

[24] Maihöfner C., Handwerker H. O., Neundörfer B., Birklein F. (2004). Cortical reorganization during recovery from complex regional pain syndrome. *Neurology*.

[25] Lloyd D., Findlay G., Roberts N., Nurmikko T. (2008). Differences in low back pain behavior are reflected in the cerebral response to tactile stimulation of the lower back. *Spine*.

[26] Pleger B., Tegenthoff M., Ragert P. (2005). Sensorimotor returning in complex regional pain syndrome parallels pain reduction. *Annals of Neurology*.

[27] Cashin A. G., McAuley J. H. (2017). Measuring two-point discrimination threshold with a caliper. *Journal of Physiotherapy*.

[28] Zimney K., Dendinger G., Engel M., Mitzel J. (2020). Comparison of reliability and efficiency of two modified two-point discrimination tests and two-point estimation tactile acuity test. *Physiotherapy Theory and Practice*.

[29] Adamczyk W. M., Luedtke K., Saulicz O., Saulicz E. (2018). Sensory dissociation in chronic low back pain: two case reports. *Physiotherapy Theory and Practice*.

[30] Fan S., Hu Z., Hong H., Zhao F. (2012). Cross-cultural adaptation and validation of simplified Chinese version of the Roland-Morris Disability Questionnaire. *Spine*.

[31] Yao M., Wang Q., Li Z. (2016). A systematic review of cross-cultural adaptation of the Oswestry disability index. *Spine*.

[32] Chiarotto A., Maxwell L. J., Terwee C. B., Wells G. A., Tugwell P., Ostelo R. W. (2016). Roland-morris disability questionnaire and Oswestry disability index: which has better measurement properties for measuring physical functioning in nonspecific low back pain? Systematic review and meta-analysis. *Physical Therapy*.

[33] Ansai J. H., Aurichio T. R., Rebelatto J. R. (2016). Relationship between dual task walking, cognition, and depression in oldest old people. *International Psychogeriatrics*.

[34] Cohen J., Cohen J. (1992). *Quantitative Methods in Psycolology: A Power Primer*.

[35] Falling C., Mani R. (2016). Ageing and obesity indices influences the tactile acuity of the low back regions: a cross-sectional study. *Manual Therapy*.

[36] Stevens J. C., Alvarez-Reeves M., Dipietro L., Mack G. W., Green B. G. (2003). Decline of tactile acuity in aging: a study of body site, blood flow, and lifetime habits of smoking and physical activity. *Somatosensory & Motor Research*.

[37] Kalisch T., Tegenthoff M., Dinse H. R. (2008). Improvement of sensorimotor functions in old age by passive sensory stimulation. *Clinical Interventions in Aging*.

[38] Lenz M., Tegenthoff M., Kohlhaas K. (2012). Increased excitability of somatosensory cortex in aged humans is associated with impaired tactile acuity. *Journal of Neuroscience*.

[39] Adamczyk W. M., Saulicz O., Saulicz E., Luedtke K. (2018). Tactile acuity (dys)function in acute nociceptive low back pain: a double-blind experiment. *Pain*.

[40] Nishigami T., Mibu A., Tanaka K. (2017). Development and psychometric properties of knee-specific body-perception questionnaire in people with knee osteoarthritis: the Fremantle Knee Awareness Questionnaire. *PLoS One*.

[41] Moseley L. G., Zalucki N. M., Wiech K. (2008). Tactile discrimination, but not tactile stimulation alone, reduces chronic limb pain. *Pain*.

[42] Apkarian A. V., Stea R. A., Bolanowski S. J. (1994). Heat-induced pain diminishes vibrotactile perception: a touch gate. *Somatosensory & Motor Research*.

[43] Flor H., Denke C., Schaefer M., Grüsser S. (2001). Effect of sensory discrimination training on cortical reorganisation and phantom limb pain. *Lancet*.

[44] Tremblay F., Wong K., Sanderson R., Coté L. (2003). Tactile spatial acuity in elderly persons: assessment with grating domes and relationship with manual dexterity. *Somatosensory & Motor Research*.

[45] Meyer S., Karttunen A. H., Thijs V., Feys H., Verheyden G. (2014). How do somatosensory deficits in the arm and hand relate to upper limb impairment, activity, and participation problems after stroke? A systematic review. *Physical Therapy*.

[46] Magni N. E., McNair P. J., Rice D. A. (2018). Sensorimotor performance and function in people with osteoarthritis of the hand: a case-control comparison. *Seminars in Arthritis and Rheumatism*.

